# Strengths and challenges of the artificial intelligence in the assessment of dense breasts

**DOI:** 10.1259/bjro.20220018

**Published:** 2022-08-11

**Authors:** Sahar Mansour, Somia Soliman, Abisha Kansakar, Ahmed Marey, Christiane Hunold, Mennatallah Mohamed Hanafy

**Affiliations:** 1 Radiology, Kasr ElAiny Hospital, Cairo University, Cairo, Egypt; 2 Baheya Foundation for Early Detection and Treatment of Breast Cancer, Giza, Egypt; 3 Pathology, Kasr ElAiny Hospital, Cairo University, Cairo, Egypt; 4 FUJIFILM Medical Imaging and IT Solutions MEA, Dubai, United Arab Emirates

## Abstract

**Objectives:**

High breast density is a risk factor for breast cancer and overlapping of glandular tissue can mask lesions thus lowering mammographic sensitivity. Also, dense breasts are more vulnerable to increase recall rate and false-positive results. New generations of artificial intelligence (AI) have been introduced to the realm of mammography. We aimed to assess the strengths and challenges of adopting artificial intelligence in reading mammograms of dense breasts.

**Methods:**

This study included 6600 mammograms of dense patterns “c” and “d” and presented 4061 breast abnormalities. All the patients were subjected to full-field digital mammography, breast ultrasound, and their mammographic images were scanned by AI software (Lunit INSIGHT MMG).

**Results:**

Diagnostic indices of the sono-mammography: a sensitivity of 98.71%, a specificity of 88.04%, a positive-predictive value of 80.16%, a negative-predictive value of 99.29%, and a diagnostic accuracy of 91.5%. AI-aided mammograms presented sensitivity of 88.29%, a specificity of 96.34%, a positive-predictive value of 92.2%, a negative-predictive value of 94.4%, and a diagnostic accuracy of 94.5% in its ability to read dense mammograms

**Conclusion:**

Dense breasts scanned with AI showed a notable reduction of mammographic misdiagnosis. Knowledge of such software challenges would enhance its application as a decision support tool to mammography in the diagnosis of cancer.

**Advances in knowledge:**

Dense breast is challenging for radiologists and renders low sensitivity mammogram. Mammogram scanned by AI could be used to overcome such limitation, enhance the discrimination between benign and malignant breast abnormalities and the early detection of breast cancer.

## Introduction

Breast cancer is the most common cancer in females and a primary cause of death from cancer, but early detection and treatment can considerably improve outcomes.^1,2^

Breast density is a radiologic term that refers to the proportion of radio-opaque parenchymal tissue in mammograms compared to the radiolucent fatty tissue.^3^ Breast density is mostly inherited, but it can be influenced by other factors throughout a female’s life. Multiple studies on dense breasts found that age, hormone use, body mass index, and the reproductive status are factors that could influence breast tissue density.^4^

High breast density is an independent risk factor and the strongest marker of breast cancer, in both premenopausal and postmenopausal females.^3^ Females with extremely dense breasts were 17 times more likely to develop interval cancer than females with fatty breasts.^5^

Dense breasts on mammogram present more false-negative results than less dense patterns. It is attributed to the masking effect that lowers the mammographic sensitivity with consequent threefold increase in the recall rate.^6^

Breast ultrasound has been used to characterize breast lesions for many years, and it has the advantage of being readily available, affordable, and well tolerated by females.^7^ It improves the sensitivity and specificity of mammography in dense breasts, elevates rates of breast cancer detection, and reduces unnecessary biopsies.^8^

In the last couple of years, mature products of artificial intelligence (AI) for computer interpretation of mammographic images have been developed and solved the limitations of the conventional computer-aided diagnosis (CAD) systems, which is the need for programmer-specified characteristics of malignancy.^9,10^

Since high density breasts are challenging and missing cancers on mammogram in such situation are not uncommon, so, we aimed to assess the impact of integrating AI with the mammogram to upgrade the diagnostic performance of cancer in high density breasts (*i.e.,* ACR c and d) in correlation with the performance of mammogram combined by breast ultrasound. The strengths and challenges that may face the reader in such situation will be discussed to enhance and support the radiologist-artificial intelligence verification of the cases diagnosis.

## Methods

This prospective study included a total of 3300 patients presented with 6600 mammograms during the period from February 2020 till February 2022. The age of the patients ranged from 29 to 83 years (mean age 47.89 ± 10.99).

All the involved patients performed full-field digital mammography, breast ultrasound. Images of mammogram were exported and processed by a diagnostic supportive AI software (Lunit INSIGHT MMG).

Confirmation of the final diagnosis was done via: 1) true cut tissue core biopsy by a 14 G needle for all suspicious/malignant abnormalities and for benign looking lesions on request of the patient /the referring physician. 2) At least 1 year of imaging follow-up by mammogram, ultrasound, and AI of stationary findings for the benign looking abnormalities and normal-looking mammograms.

The study was approved by the ethical committee of the Radiology department, and an informed written consent was taken from all patients.

### Inclusion criteria

- Patients with dense breasts on a mammogram (ACR c or d) presented for either screening or diagnostic scanning.

### Exclusion criteria

- Patients with low breast density on a mammogram (ACR a or b).
- Patients with induced increased density of the breast whether due to chemotherapy or recent post-operative changes.
- Patients with a contraindicated status for mammography, *e.g.* pregnancy.

### Equipment

- Full-field digital mammography machine (Amulet Innovality, Fujifilm Global Company, Japan)
- Ultrasound device (LOGIQ S8-GE) using a high-frequency linear probe (7–12 MHz) for breast scanning.
- AI software (Lunit INSIGHT MMG ver. 1.10.2, Seoul, South Korea, FDA approved, version 2019) provided by AI algorithm for scanning and reading mammograms.

### Image analysis and interpretation

Breast density was assessed for each patient according to ACR Mammography “Breast Imaging Reporting and Data System” BI-RADS atlas 2013; ACR c (the breasts are heterogeneously dense) and ACR d (the breasts are extremely dense).

The average mammographic density provided by the AI-based computer-assisted diagnosis (AI-CAD) program of the standard four mammographic views with a 1–10 scale: density A was defined with scores 1 to 2, B with scores 3 to 5, C with scores 6 to 8, and D with scores 9 to 10.^11^

Interpretation of mammogram and ultrasound was done following the standard reporting system in BI-RADS ACR atlas fifth edition 2013,^12^ for 4061 mammograms that showed benign and malignant breast abnormalities. Breast with multiple masses was assigned by the category of the mass presented the highest BI-RADS category score.

The used AI algorithm was developed based on deep convolutional neural networks (CNNs). ResNet-34, which is one of the most popular CNN architectures, was used as a backbone network.^13^

The algorithm training consisted of two stages: patch-level training from scratch for learning low-level features (Stage 1), followed by image-level finetuning from the stage-1 model for learning high-level context (Stage 2). For each mammogram view (*i.e.,* one of the four traditional bilateral cranio-caudal and medio-lateral oblique views), the AI algorithm provides pixel-level abnormality scores as a heatmap and representative abnormality numerical scores which are floating-point values between 0 and 1.

The heat map highlighted the breast abnormality, provided locations information and generated scoring percentage reflecting the probability of malignancy for the lesion within the range <10 and 100% (100% represents the highest level of suspicion).

The AI category was determined for each breast according to the probability of malignancy score as follows: 0–9 for definite non-cancer, 10–25 for probably non-cancer, 26–50 for possibly non-cancer, 51–75 for possibly cancer, 76–99 for probably cancer, and 100 for definite cancer.^14^

Correlation between the sono-mammographic BI-RADS and the AI category for each breast and the histopathological results or follow-up were performed.

### Statistical analysis

Data were coded and entered using the statistical package SPSS (Statistical Package for the Social Sciences) version 26 (IBM Corp., Armonk, NY, USA). Data were summarized using mean, standard deviation, minimum, and maximum in quantitative data and using frequency (count) and relative frequency (percentage) for categorical data. Standard diagnostic indices including sensitivity, specificity, positive-predictive value (PPV), negative-predictive value (NPV), and diagnostic efficacy were calculated. For comparing categorical data, the chi-square (c2) test was performed. Exact test was used instead when the expected frequency is less than 5.

The interobserver variability was measured (to calculate measurement error intrinsic to between-observer difference) using κ indices. Confidence interval percentage (CI %) was done for the range of the abnormality scoring values elicited by the AI software where the narrower the interval (upper and lower values), the more precise is the AI estimate.

The highest area under the ROC curve was plotted for the AI performance when added to the mammogram and a cut off value was calculated for the abnormality scoring percentage.

## Results

In the current study, 3135/3300 (95 %) cases were assigned a breast density score of “c” and 165/3300 (5%) cases were assigned a score of “d” according to the ACR breast density classification.

Unilateral lesions presented in 1867 (56.5%) cases and 1097 cases (33.4%) showed bilateral breast lesions and 336 cases showed bilateral normal mammograms (10.1%)

Normal “BI-RADS one category” was assigned for 2539 mammograms. There were 4061 mammograms with breast abnormalities:1892 benign (46.6%) and 2169 malignant (53.4%). True-cut tissue biopsy was used to confirm the diagnosis of all the malignant lesions (*n* = 2169) and 14% (*n* = 569) of the benign masses. The remaining of the included benign lesions; 32.6% (*n* = 1327) and the normal assigned mammograms were confirmed by stationary course after one year of follow-up with regard conventional imaging (mammogram and ultrasound) and AI scoring of <10%.

The malignant group included: invasive ductal carcinoma which was the commonest histopathological type {76% (*n* = 1646/2169)}, followed by invasive lobular carcinoma {10.65%, (*n* = 231/2169)}, ductal carcinoma *in-situ* {3.9%, (*n* = 85/2169)}, combined invasive ductal and invasive lobular carcinoma {3.6%, *n* = 79/2169), mucinous carcinoma{2.4%, *n* = 52/2169), papillary carcinoma{2.1%, *n* = 47/2169), tubular carcinoma {0.5%, (*n* = 10/2169)}, medullary carcinoma{0.3%, *n* = 7/2169), malignant phyllodes{0.3% *n* = 7/2169), micropapillary carcinoma {0.2%, *n* = 4/2169)}, and metaplastic carcinoma {0.05%, *n* = 1/2169)).

The distribution of the different pathological entities was demonstrated in Table 1

**Table 1.** The distribution of the included pathological entities

Final diagnosis (*n* = 4061)		Count (n)	%
**Malignant** **(*n* = 2169, 53.4%)**	Invasive ductal carcinoma	1646	76%
	Invasive lobular carcinoma	231	10.65%
	Ductal carcinoma *in situ*	85	3.9%
	Combined invasive ductal and invasive lobular carcinoma	79	3.6%
	Mucinous carcinoma	52	2.4%
	Papillary carcinoma	47	2.1%
	Tubular carcinoma	10	10%
	Medullary carcinoma	7	0.3%
	Malignant phyllodes	7	0.3%
	Micropapillary carcinoma	4	0.2%
	Metaplastic carcinoma	1	0.05%
**Benign** **(*n* = 1892, 46.6%)**	Simple fibroadenoma	1226	64.8%
	Atypical ductal hyperplasia	111	5.9%
	Epithelial hyperplasia	75	4%
	Proliferative fibrocystic mastopathy	105	5.5%
	Benign phyllodes	37	2%
	Intraductal papilloma	34	1.8%
	Granulomatous mastitis	34	1.8%
	Pseudoangiomatous stromal hyperplasia (PASH)	32	1.7%
	Complex adenoma	30	1.6%
	Sclerosing adenosis	24	1.2%
	Adenosis with no malignancy detected	124	6.5%
	Fat necrosis	55	2.9%
	Pleomorphic adenomas	5	0.3%

The included benign and malignant breast abnormalities were either mammography detected or mammography indiscernible from the overlapping glandular tissue and were detected on ultrasound scanning.

BI-RADS category was given according to the combined evaluation of the mammography and ultrasound where 1544 breast lesions (23.4 %) were given BI-RADS 5 and 1125 lesions (17.1%) were given BI-RADS 4.

BI-RADS 3 were assigned for 698 breast lesions (10.6%), BI-RADS 2 for 694 lesions (10.5%), and BI-RADS 1 for 2539 mammograms/ultrasound (38.4%).

The mammography and ultrasound findings of the studied breast abnormalities (total = 4061) were demonstrated in Table 2.

**Table 2.** Mammography and ultrasound findings among the studied breast abnormalities (P.S. each mammogram/ultrasound may include more than one finding in relation to the same disease)

Mammography Findings	Count(n)	%
Mass	2359	58.1%
Asymmetry	629	15.5%
Distortion	495	12.2%
Benign calcifications	410	10.1%
Malignant calcifications	849	20.9%
**Ultrasound Findings**	**Count(n)**	**%**
Solid	2745	67.6%
Cystic	739	18.2%
Complex	357	8.8%
Altered parenchyma	109	2.7%
Distortion	81	2.0%
Interstitial edema ±skin thickening	438	10.8%

For statistical purposes, BI-RADS 4 and 5 assigned breast abnormalities (*n* = 2669/6600) were considered cancer positive mammograms, while BI-RADS categories 1, 2, and 3 were considered negative for malignancy (*n* = 3931/6600).

After revising results of pathology or close follow-up (for benign looking or normal cases), 2141 mammograms were true positive, 3901 were true negative, 530 were false positive, and 28 were false negative.

Diagnostic indices of the performance of the mammogram combined by the ultrasound without consideration of the AI scoring were a sensitivity of 98.71% (95% CI: 93.13 to 99.95%), a specificity of 88.04% (95% CI:75.87 to 98.34%), a positive likelihood ratio of 1.1341 (95% CI: 0.0741 to 2.6541), a negative likelihood ratio of 1.1098 (95% CI: 1.0898 to 1.2198), a PPV of 80.16% (95% CI: 73.49 to 85.53%), a NPV of 99.29% (95% CI: 88.71 to 99.99%), and a diagnostic accuracy of 91.5% (95 % CI: 84.72 to 96.59%).

AI applied scoring and category of the studied breast abnormalities were presented by Table 3

**Table 3.** Percentage and category of each of the AI-scanned mammograms for the included benign and malignant lesions

Artificial intelligence category/scoring		Count(n)	%
**Definite cancer**	100%	9	0.2%
**Probably cancer**	76–99%	1637	40.3%
**Possibly cancer**	51–75%	431	10.6%
**Possibly non-cancer**	26–50%	956	23.5%
**Probably non-cancer**	10–25%	734	18%
**Definite non-cancer**	Low (0–9%)	294	7.2%

The scoring of the AI software was correlated with the pathology, where 1915 mammograms showed true-positive abnormalities, 4269 mammograms were true negative (*n* = 1730 benign and *n* = 2539 normal) ,162 were false positive, and 254 were false negative. So, the evaluation of the AI algorithm presented a sensitivity of 88.29% (95% CI: 78.63 to 95.05%), a specificity of 96.34% (95% CI: 87.07 to 99.98%), a positive likelihood ratio of 0.9261 (95% CI: 0.853 to 1.239), a negative likelihood ratio of 0.9061 (95% CI: 0.8461 to 1.0261), a PPV of 92.2% (95% CI: 84.31 to 96.38%) , a NPV of 94.4% (95% CI: 85.97 to 99.98%), and a diagnostic accuracy of 94.5% (95% CI: 88.24 to 99.15%) in its ability to read dense mammograms.

The diagnostic indices of the performance of the mammogram and ultrasound with and without the consideration of the AI scoring were summarized in Table 4.

**Table 4.** Diagnostic indices of the performance of the mammogram and ultrasound with and without the consideration of the AI scoring

Diagnostic indices	Sensitivity	Specificity	+ve LHR	-ve LHR	PPV	NPV	Accuracy
**Sono-mammogram without AI**	98.71%	88.04%	1.1341	1.1098	80.16%	99.29%	91.5%
**AI integrated with sono-mammogram**	88.29	96.34%	0.9261	0.9061	92.2%	94.4%	94.5%

LHR, Likelihood ratio; NPV, Negative predictive value; PPV, Positive predictive value.

The highest area under the ROC curve was plotted for the AI performance when added to the mammogram and ultrasound to be 0.934 (95% CI: 0.854 to 0.966) which correlated with a cut-off value of 39% as the optimal abnormality scoring percentage to distinguish benign from malignant breast abnormalities in dense breasts.

Reading of the included breast abnormalities was enhanced after the consideration of the abnormality scoring of the AI algorithm in the final diagnosis: from a κ value of 0.86 (95% CI, 0.77–0.93) for conventional imaging to 0.91 (95% CI, 0.90–0.92) for the added AI scoring to the radiologic diagnosis.

## Discussion

^11^ Higher mammographic breast density may adversely affect the sensitivity and specificity of mammography.^15^

With a relatively low number of expert screening radiologists, the expansion of breast cancer screening programs necessitates assistance in mammography evaluation to increase the detection rate and reduce workload issues. AI in breast cancer screening may meet these needs and can be used as a second reader for mammographic images.^16,17^

The diagnostic performance of AI was less affected by breast density than the performance of radiologists, resulting in a significant improvement in radiologists' AI-aided performance in dense breasts.^3^

This study discusses the performance of AI in the detection and establishment of the correct diagnosis for mammograms with high breast density *i.e.,* ACR “c” and “d”.

The findings of sono-mammography and the AI system were analyzed in 3300 patients, their ages ranged between 29 and 83 years with a mean age of 47.89 ± 10.99, and all had primary dense breasts.

Previous work found females of mean age 44 ± 7 years had the highest breast density with a significant negative correlation observed between age and breast density category.^18^

Among the malignant group, invasive ductal carcinoma (IDC) was the commonest histopathological type {76%(*n* = 1646)} and simple fibroadenoma {64.8% (*n* = 1226)} was the commonest benign one.

The study included 530 false-positive diagnosed lesions by sono-mammogram that included: 111 atypical ductal hyperplasia, 75 epithelial hyperplasia, 67 proliferative fibrocystic mastopathy, 46 simple fibroadenomas, 37 benign phyllodes, 34 intraductal papilloma, 34 granulomatous mastitis, 32 pseudoangiomatous stromal hyperplasia (PASH), 30 complex adenoma, 24 sclerosing adenosis, 18 adenosis with no malignancy detected, 17 fat necrosis, and five pleomorphic adenomas.

False-negatives were encountered in 28 lesions were proved to be 1ten non-calcified DCIS, seven lobular carcinoma, five mucinous carcinoma, two papillary carcinoma, two malignant phyllodes, one metaplastic carcinoma, and one medullary carcinoma by pathological diagnosis.

Thus, the elicited sensitivity was 98.71% and specificity was 88.04% for sono-mammography. Chen et al, found out that the sensitivity of mammography was significantly low in patients with high breast density where it showed 84.5% in low breast density verses 65.8% in case of dense breasts (*p* < .001). Also, they stated that ultrasound had higher sensitivity than mammography (*p* < .001), and a better sensitivity was achieved when mammography was combined with ultrasound than mammography or ultrasound alone (*p* < .001),^19^

Previous studies have shown that using AI supports the decision of the radiologists during their work in assessing breast lesions.^17^

AI-based CAD characterizes the detected lesion and stratifies the risk of biopsy. This would increase the positive-predictive value of biopsy while decreasing intra- and interobserver variation by reducing the impact of AI training and experience differences.^20^

In this experience, reading of the mammograms was enhanced from a κ value of 0.86 to 0.91 (95% CI, 0.90–0.92) when supported with the AI algorithm.

An AI abnormality scoring, and category was given to each breast; 2077/6600 (31.5 %) breast lesions were considered malignant (AI scoring and categories: 51–75 possibly cancer, 76–99 probably cancer, 100 definite cancer) and 4523/6600 (68.5 %) breast lesions were considered benign (AI scoring and categories: 0 definite non-cancer, 1–25 probably non-cancer, 26–50 possibly non-cancer) where 2539 out of them presented normal mammograms of “Low” scoring value (i.e. <10%).

**Figure 1.**
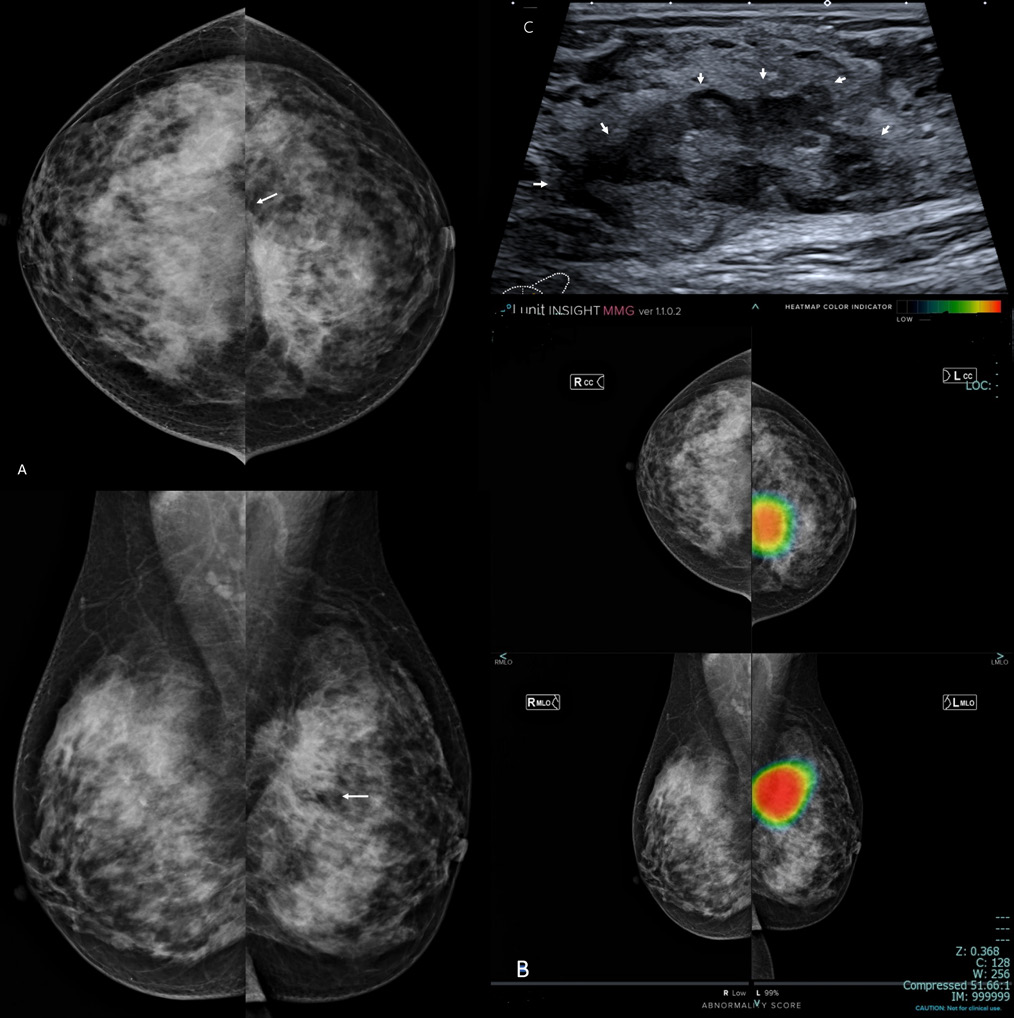
Left breast invasive ductal carcinoma grade III in a female patient 37-years-old with extremely dense breasts (ACR d). A: bilateral digital mammogram (cranio-caudal and medio-lateral oblique views). The density of the breasts decreased the mammogram sensitivity- there is no obvious masses at first sight- the left breast showed upper inner tissue peaking (arrow) which is indirect suspicion sign of malignancy. B: AI scanned mammogram presented intense color hue that targeted specifically the left breast mass, confirmed the suspicion of malignancy in the form of abnormality scoring of 99% and displayed the full extent of the disease. C: Ultrasound image displayed comparable information to that of the AI where there was 11 clock left breast purely solid irregular mass (BI-RADS 5) (arrows). The case was a true positive on the level of conventional imaging and AI algorithm

**Figure 2.**
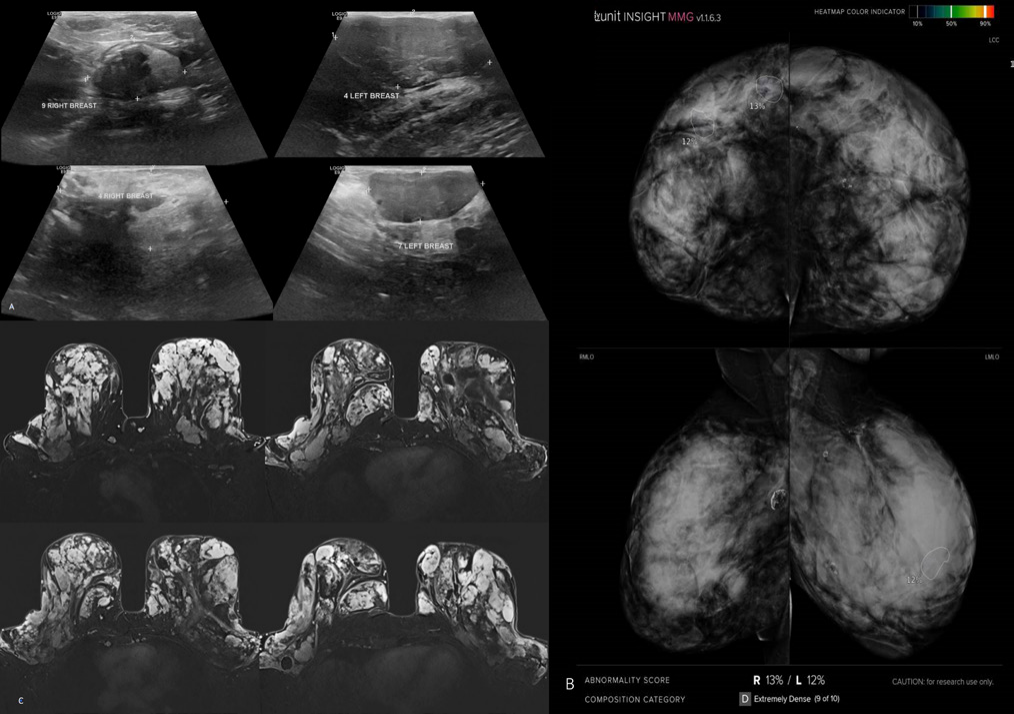
Idiopathic gigantomastia of a 34-year-old female with extremely dense breasts (ACR d). A, Ultrasound images of both breasts showed multiple solid masses, some of them showed indeterminate features of heterogeneous texture and lobulated outline (BI-RADS 3). B: four view mammograms scanned by the AI algorithm showed low scoring of malignancy at both breasts (right, 13% and left, 12%). C, MR examination, axial subtraction post contrast images; showed both breasts studded by multiple variable-sized solid masses characteristic of adenomas. Biopsy from the ones that showed high vascularity revealed simple fibroadenoma. The case was a true negative for AI

In our study, the overall accuracy of AI (94.5%) was higher than that of combined mammography and ultrasound (91.5%). Upon correlation with the pathology or the interval follow-up, there were 1915 true positive, Figure 1 and 1730 true benign abnormalities, Figure 2.

Our results were comparable to a multireader study conducted upon 2652 exams and interpreted by 101 radiologists versus the AI software. Their study showed that the diagnostic accuracy of the AI system was statistically non-inferior to that of the radiologists. The AI system had a diagnostic accuracy of 84% and the average of the radiologists was 81.4%.^21^

**Figure 3.**
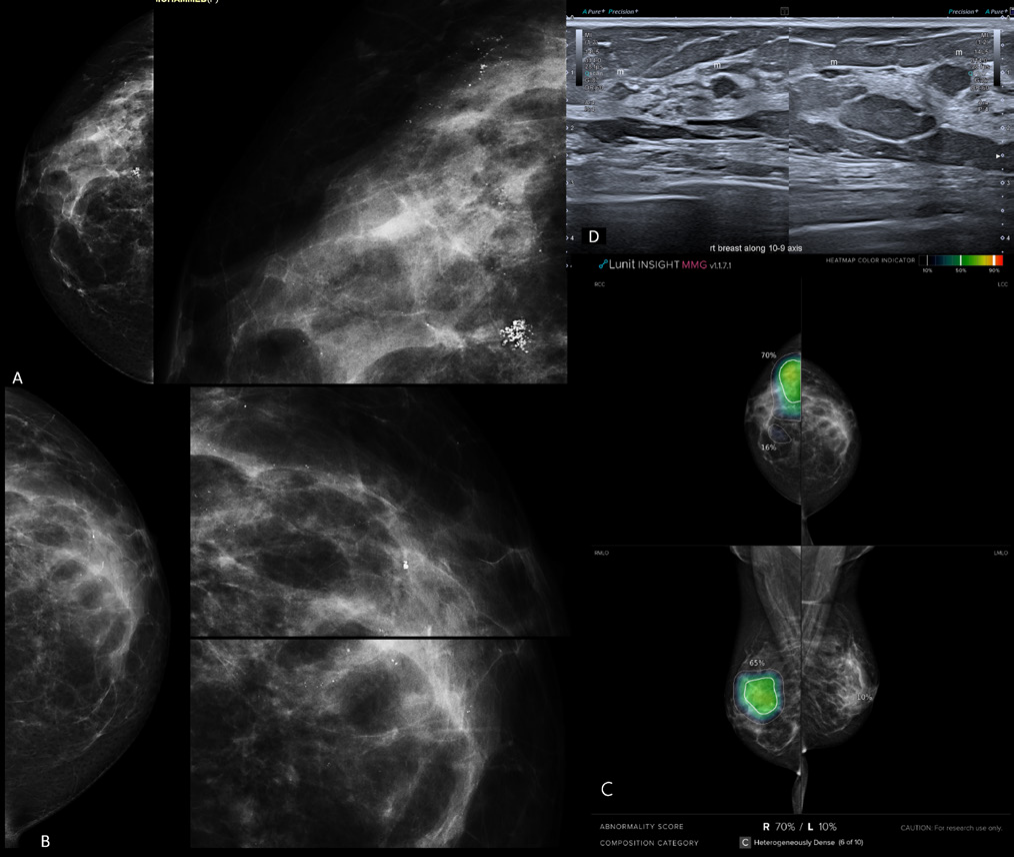
Heterogeneously dense breasts (ACR c); right multifocal invasive ductal carcinoma and left fibrocystic changes. A, digital mammogram of the right breast (whole breast and magnification view of the area of interest) showed deep central focal clustered pleomorphic and upper outer segmental suspicious micro calcifications. B, digital mammogram of the left breast (whole breast and magnification view of the areas of interest) showed upper outer and deep central segmental indeterminate micro calcifications. C, the AI algorithm outlined not just the clustered pleomorphic calcifications yet also included the upper outer segmental ones with the same degree of colour hue intensity and gave the whole area high abnormality scoring percentage of 70 %. On the left side there was low scoring percentage of 10% (definite not cancer) that was matching with the benign pathology of fibrocystic changes. D, Ultrasound image that showed small mass in correlation with the clustered calcifications. Additional tiny masses were found along the line of distribution of the segmental calcifications guided by the AI targeted color hue image. The case was a right true positive and a left true negative.

The radiologist could use AI-CAD in the characterization of the suspicious microcalcifications on mammography as its performance was similar to that of the radiologists where the estimated area under the curves (AUCs) of the radiologists and AI-CAD were not significantly different (0.722 *vs* 0.745) which indicated that AI-CAD could be of benefit in making clinical decisions for breast microcalcification and accordingly avoid unnecessary biopsies.^22^ Through our experience, it was noted that the extend of the color hue and the abnormality scoring could be a clue to distinguish suspicious microcalcifications from the overlapping grouped benign ones (Figure 3).

It was also observed that although the AI presented lower sensitivity than sono-mammography in correlation with the final diagnosis, yet the specificity was higher. These results were comparable to another study performed by Roela et al., 2021 ^23^ **.** Their study showed that the sensitivity of AI; AI with radiologist; radiologist alone, was 76.08%; 84.02%; 80.91%, respectively. Specificity for AI; AI with radiologist; radiologist alone, was 96.62%; 85.67%; 84.89% respectively.^23^ Mansour and co-authors^24^ showed a sensitivity of 96.8% and a specificity of 90.1% in the discrimination between benign and malignant breast lesions by the AI-aided mammograms and mammography combined with ultrasound examinations showed a sensitivity of 98.6% and a specificity of 91.6%.

Some previous work had focused on the stand-alone performance of AI and compared it to the radiologist. For instance, Rodriguez-Ruiz, et al^21^ found that the AI system had AUC higher than 61.4% of the radiologists since that the average value of the AI system was a 0.840 (95% CI: 0.820 to 0.860) and the average of the radiologists was 0.814.

Further studies showed the performance level of AI at the mammogram reader level was 0·940,^25^ and when combined the AI algorithms with the ultrasound, performed by the radiologist, the higher area under the curve was 0.942 and achieved a significant specificity and sensitivity of 92.0%.

The current work agreed with these studies where the addition of the AI to the performance of the mammogram and ultrasound showed the highest point for area under the ROC to be 0.934 (95% CI: 0.854 to 0.966), which achieved higher specificity (96.34%) than the performance of the included imaging modalities without AI (88.04%) which correlated with a cut-off value of 39% in discrimination between benign and malignant abnormalities at dense breasts.

There were 162 false-positive lesions by AI in the current work. The relative decrease in false-positive lesions by AI compared to sono-mammography is contributed to the fact that the AI system performs two separate tasks. It does not only detect lesions but also classify them. For example, masses are classified according to density (gray level), shape, texture, and relation to surrounding to reach a final diagnosis.^16^

False-positive pathologies were proliferative fibrocystic mastopathy with micro-calcifications (*n* = 45), adenosis with calcifications (*n* = 40), fibrocystic changes without calcifications (*n* = 30), granulomatous mastitis (*n* = 21), benign phyllodes (*n* = 10), fat necrosis (*n* = 7), intraductal papilloma (*n* = 3), sclerosing adenosis (*n* = 3) pseudoangiomatous stromal hyperplasia (*n* = 2), and adenosis without calcifications (*n* = 1).

**Figure 4.**
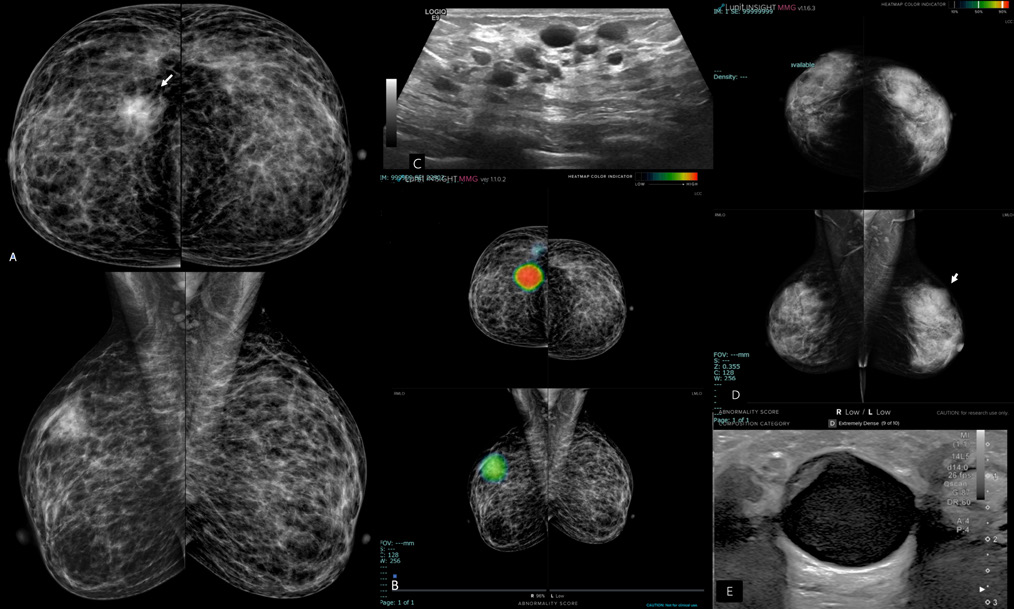
Two cases; 4A, 4B and 4C, right breast atypical ductal hyperplasia in a heterogeneously dense breasts of a 37-year-old female (ACR c). A: bilateral mammograms showed right breast upper outer quadrant indistinct high dense abnormality likely mass (arrow). B, mammograms scanned by the AI algorithm displayed localized intense red color of the detected right breast abnormality and a very high abnormality scoring of 96% suggestive of most likely carcinoma. C, Ultrasound image of the right breast abnormality presented a cluster of small slightly turbid cysts (BI-RADS 3). The case with the aid of ultrasound was a true negative while it was a false positive for AI. Second case; 4D and 4E, female patient 50-year-old with dense breasts (ACR d), that had a left breast upper outer quadrant complicated large cyst. D: Artificial intelligence -aided mammograms displayed “Low” scoring although there is an obvious left breast upper outer quadrant mass of partly circumscribed, partly obscured border (arrow). E, ultrasound image of the left breast showed large cystic mass with turbid echogenic content and mural based non-soft tissue solid component

Incorrect high scoring in case of benign conditions presented in mammograms with heterogenous and/or in-distinction abnormalities as in extensive fibrocystic changes and diffuse scattered heavy powdery secretory-induced calcifications. Also small, localized organization of small, clustered cysts may mimic the AI algorithm of irregular dense mass on mammogram (Figure 4a, b c). However, in case of unifocal cyst, the AI algorithm showed specific scoring and suggested low percentage “Low” scoring i.e.,<10% suspicion of malignancy irrespective of the size and/or inhomogeneous internal texture of the breast lesion (Figure 4d e.)

**Figure 5.**
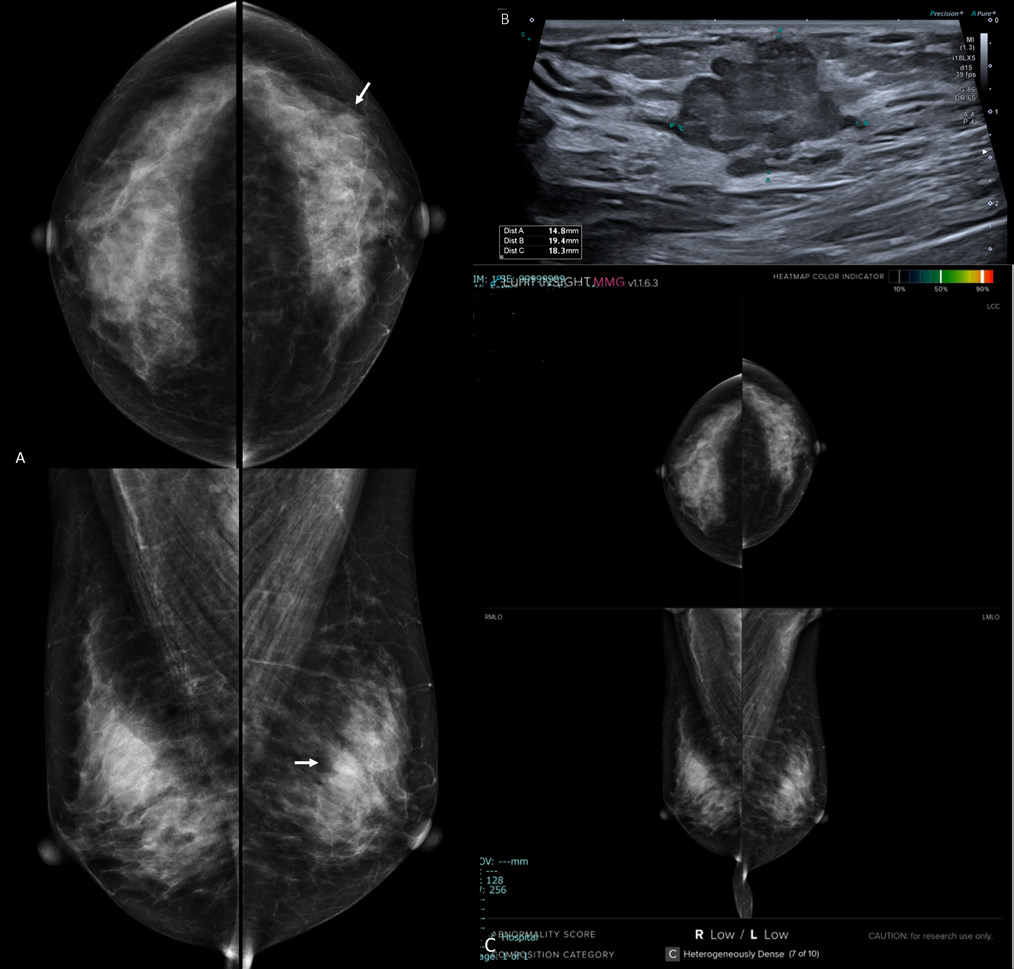
Female patient 50 years old with heterogeneously dense breasts (ACR c) and diagnosed by left breast mucinous carcinoma grade I. A: bilateral digital mammogram (cranio-caudal and medio-lateral oblique views). There is a left breast upper outer suspected mass predominantly obscured by glandular tissue (arrow). B: Ultrasound image displayed irregular solid mass of fat near echopattern and in plane with the breast tissue. C: Artificial intelligence scanned 4 view mammograms displayed no visual color overlying and “Low” (i.e., < 10%) abnormality scoring suspicion of malignancy

**Figure 6.**
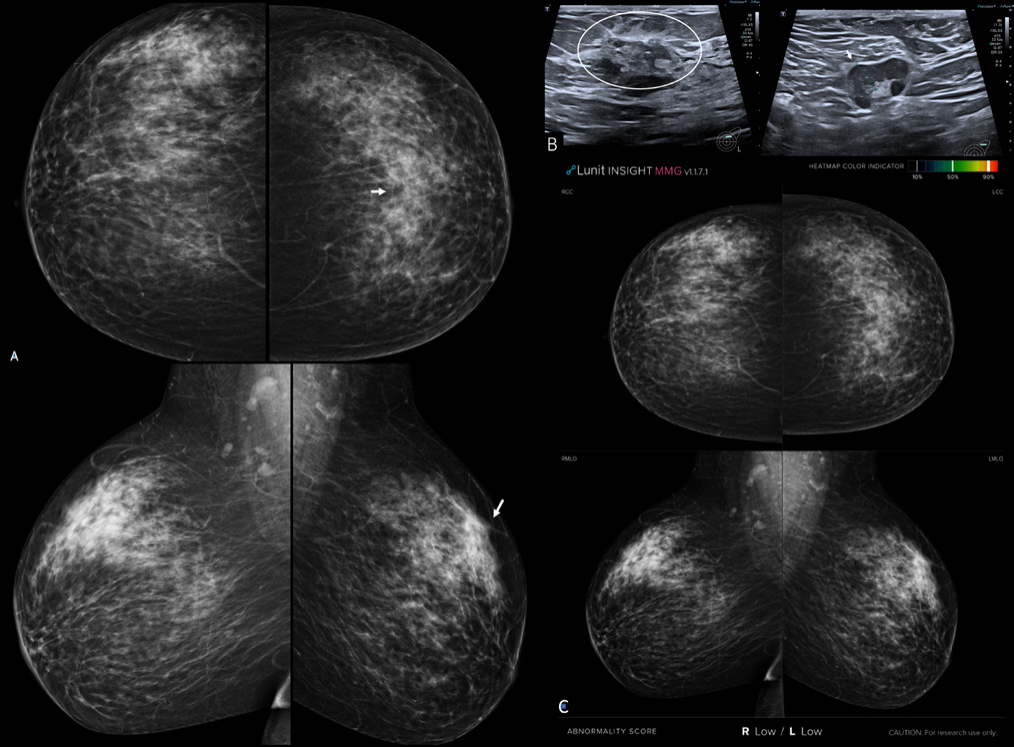
Left invasive lobular carcinoma in heterogeneously dense breasts (ACR c). A: bilateral mammograms showed indirect sign of malignancy presented by tissue peaking (arrow) seen at the left breast upper central region. B, Ultrasound image that targeted the left breast abnormality showed indistinct isoechoic solid mass and in plane with the breast tissue (BI-RADS 4). C, AI- scanned mammograms showed “Low” scoring not matching with the mammogram and the ultrasound findings. The left breast was a false negative count by AI.

On the other side, there were 254 false-negative lesions that was skipped from the AI algorithm due to either lesions were of small size, lesions were with relative fat near density**,** or both criteria (Figure 5)**.** Also among the false-negative challenges were masses that showed breaking down with relatively decreased mammographic density as well as pathologies that presented by just asymmetry or increased density that displayed pattern of extension in plane with the breast tissue and were associated with no obvious distortion on mammogram, Figure 6.

False-negative results of AI, in general, may be attributed to the fact that it depends on the extraction of quantitative features, these features are related to tumor size, shape, intensity, and texture to depict information on pixel distribution within the image and provide a comprehensive results characterization.^26,27^

As AI algorithms become more complex, AI may begin to serve as an assistant rather than a tool, occasionally it can act independently with frequent periods of supervision by an expert radiologist.^28^

MG has limited diagnostic sensitivity in patients with small breast cancer, especially in those with dense breast tissue. US is better than MG at detecting small breast cancer, regardless of breast density.^20^

**Figure 7.**
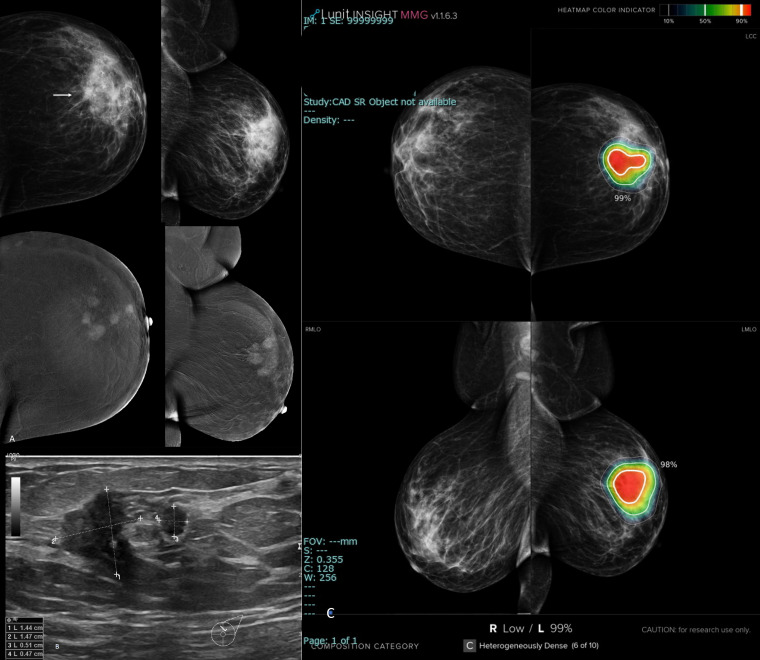
Multifocal invasive tubular/cribriform carcinoma of the left breast. A: upper row, digital mammogram craniocaudal and medio-lateral oblique views of the left breast that showed a small suspicious lesion (arrow). Lower row, contrast enhanced mammogram that showed multiple malignant looking masses not just one mass. B, Ultrasound image that showed the index malignant mass which was less than 2 cm and an adherent minion. C, AI- scanned mammograms showed wide area of hot color demarcation and an abnormality scoring percentage of 99%. In this case, the AI algorithm correctly diagnosed and targeted the carcinoma and moreover it provided similar extension of the small sized index mass and the related satellites to that of the contrast enhanced mammogram.

During the current experience, it was observed that AI can detect small-sized carcinoma and moreover the tiny minions that could be a challenge in high density breasts (Figure 7).

The used AI algorithm does not score typically benign looking masses and sometimes underestimate breast lesions that lack most of the mammographic criteria of malignancy. In such situation, no focal areas will be spotted by the color hue at the heatmap images and the given abnormality scoring for the breast as a whole will be “Low” scoring.

With the AI assistnace, breast abnormalities in densely glandular tissue are easily targeted, probably categorized in less time and effort for tissue diagnosis. However,to get the best performance of AI-aided mammograms in case of dense breast (*i.e.,* ACR c and d) and overcome the possibility of mismatching low scoring, it is recommended to support the AI-mammogram findings with an ultrasound examination.^24^

Also, in case of diffuse breast diseases and proved malignancy; contrast-based study (*i.e.,* contrast -enhanced MR imaging or contrast enhanced mammogram) is recommended in addition to the traditional imaging for precise disease demonstration (*i.e.,* diagnosis and extension) Figures 2 and 7.

## Conclusion

The AI algorithm applied on mammogram of the dense breasts showed a notable reduction of sono-mammographic misdiagnosis. Knowledge of such software challenges would enhance its application as a decision support tool for screening and diagnostic mammography of breasts with heavy glandular tissue.

## References

[1] Sung H, Ferlay J, Siegel RL, Laversanne M, Soerjomataram I, Jemal A, et al . Global cancer statistics 2020: GLOBOCAN estimates of incidence and mortality worldwide for 36 cancers in 185 countries. CA Cancer J Clin 2021; 71: 209–49. doi: 10.3322/caac.21660 33538338

[2] Sadoughi F, Kazemy Z, Hamedan F, Owji L, Rahmanikatigari M, Azadboni TT . Artificial intelligence methods for the diagnosis of breast cancer by image processing: a review. Breast Cancer (Dove Med Press) 2018; 10: 219–30. doi: 10.2147/BCTT.S175311 30555254 PMC6278839

[3] Kim EY, Chang Y, Ahn J, Yun J-S, Park YL, Park CH, et al . Mammographic breast density, its changes, and breast cancer risk in premenopausal and postmenopausal women. Cancer 2020; 126: 4687–96. doi: 10.1002/cncr.33138 32767699

[4] Hashemi EA, Haghighat S, Olfatbakhsh A, Tafazoli Harandi H, Beheshtian T . (n.d.). Investigating the factors affecting the mammographic density of breast tissue in patients referred to the breast cancer research center, iran. Mcij; 1: 27–31. doi: 10.21859/mci-010212

[5] Cruwys C, Pushkin J . Breast density and impacts on health. Ecancermedicalscience 2017; 11: ed70. doi: 10.3332/ecancer.2017.ed70 28900477 PMC5574657

[6] Bojanic K, Vukadin S, Sarcevic F, Malenica L, Grgic K, Smolic R, et al . Impact of breast density awareness on knowledge about breast cancer risk factors and the self-perceived risk of breast cancer. Diagnostics (Basel) 2020; 10: E496. doi: 10.3390/diagnostics10070496 PMC7399945 32698375

[7] Mendelson EB, Tobin CE . Critical pathways in using breast US. Radiographics 1995; 15: 935–45. doi: 10.1148/radiographics.15.4.7569138 7569138

[8] Azzam H, Kamal RM, Hanafy MM, Youssef A, Hashem LMB . Comparative study between contrast-enhanced mammography, tomosynthesis, and breast ultrasound as complementary techniques to mammography in dense breast parenchyma. Egypt J Radiol Nucl Med 2020; 51: 1–9. doi: 10.1186/s43055-020-00268-1

[9] Sechopoulos I, Teuwen J, Mann R . Artificial intelligence for breast cancer detection in mammography and digital breast tomosynthesis: state of the art. Semin Cancer Biol 2021; 72: 214–25. doi: 10.1016/j.semcancer.2020.06.002 32531273

[10] Sechopoulos I, Mann RM . Stand-alone artificial intelligence - the future of breast cancer screening? Breast 2020; 49: 254–60. doi: 10.1016/j.breast.2019.12.014 31927164 PMC7375643

[11] Lee SE, Son N-H, Kim MH, Kim E-K . Mammographic density assessment by artificial intelligence-based computer-assisted diagnosis: A comparison with automated volumetric assessment. J Digit Imaging 2022; 35: 173–79. doi: 10.1007/s10278-021-00555-x 35015180 PMC8921363

[12] Sickles EA, D’Orsi CJ, Bassett LW, et al . ACR BI-RADS® Mammography. Reston, VA, American College of Radiology: ACR BI-RADS® Atlas, Breast Imaging Reporting and Data System; 2013.

[13] Nikitin V, Filatov A, Bagotskaya N, Kil I, Lossev I, Losseva N . Improvement in ROC curves of readers with next generation of mammography CAD. C-2315. ECR 2014. doi: 10.1594/ecr2014/C-2315

[14] He K, Zhang X, Ren S, Sun J. Deep Residual Learning for Image Recognition . 2016 IEEE Conference on Computer Vision and Pattern Recognition (CVPR; Las Vegas, NV, USA. Vol. 1; 2016. pp. 770–78. doi: 10.1109/CVPR.2016.90

[15] Arieno A, Chan A, Destounis SV . A review of the role of augmented intelligence in breast imaging: from automated breast density assessment to risk stratification. AJR Am J Roentgenol 2019; 212: 259–70. doi: 10.2214/AJR.18.20391 30422711

[16] Geras KJ, Mann RM, Moy L . Artificial intelligence for mammography and digital breast tomosynthesis: current concepts and future perspectives. Radiology 2019; 293: 246–59. doi: 10.1148/radiol.2019182627 31549948 PMC6822772

[17] Rodríguez-Ruiz A, Krupinski E, Mordang J-J, Schilling K, Heywang-Köbrunner SH, Sechopoulos I, et al . Detection of breast cancer with mammography: effect of an artificial intelligence support system. Radiology 2019; 290: 305–14. doi: 10.1148/radiol.2018181371 30457482

[18] Kang Y-J, Ahn SK, Kim SJ, Oh H, Han J, Ko E . Relationship between mammographic density and age in the united arab emirates population. J Oncol 2019; 2019: 7351350. doi: 10.1155/2019/7351350 31467543 PMC6701291

[19] Chen H-L, Zhou J-Q, Chen Q, Deng Y-C . Comparison of the sensitivity of mammography, ultrasound, magnetic resonance imaging and combinations of these imaging modalities for the detection of small (≤2 cm) breast cancer. Medicine (Baltimore) 2021; 100(26): e26531. doi: 10.1097/MD.0000000000026531 34190189 PMC8257894

[20] Morgan MB, Mates JL . Applications of artificial intelligence in breast imaging. Radiol Clin North Am 2021; 59: 139–48. doi: 10.1016/j.rcl.2020.08.007 33222996

[21] Rodriguez-Ruiz A, Lång K, Gubern-Merida A, Broeders M, Gennaro G, Clauser P, et al . Stand-alone artificial intelligence for breast cancer detection in mammography: comparison with 101 radiologists. J Natl Cancer Inst 2019; 111: 916–22. doi: 10.1093/jnci/djy222 30834436 PMC6748773

[22] Do YA, Jang M, Yun BL, Shin SU, Kim B, Kim SM . Diagnostic performance of artificial intelligence-based computer-aided diagnosis for breast microcalcification on mammography. Diagnostics (Basel) 2021; 11(8): 1409. doi: 10.3390/diagnostics11081409 34441343 PMC8392744

[23] Roela RA, Valente GV, Shimizu C, Lopez RVM, Tucunduva T de M, Folgueira GK, et al . Deep learning algorithm performance in mammography screening: A systematic review and meta-analysis. JCO 2021; 39: e13553. doi: 10.1200/JCO.2021.39.15_suppl.e13553

[24] Mansour S, Kamal R, Hashem L, AlKalaawy B . Can artificial intelligence replace ultrasound as a complementary tool to mammogram for the diagnosis of the breast cancer? Br J Radiol 2021; 94(1128): 20210820. doi: 10.1259/bjr.20210820 34613796 PMC8631011

[25] Kim H-E, Kim HH, Han B-K, Kim KH, Han K, Nam H, et al . Changes in cancer detection and false-positive recall in mammography using artificial intelligence: a retrospective, multireader study. Lancet Digit Health 2020; 2: e138–48. doi: 10.1016/S2589-7500(20)30003-0 33334578

[26] Romeo V, Accardo G, Perillo T, Basso L, Garbino N, Nicolai E, et al . Assessment and prediction of response to neoadjuvant chemotherapy in breast cancer: A comparison of imaging modalities and future perspectives. Cancers (Basel) 2021; 13(14): 3521. doi: 10.3390/cancers13143521 34298733 PMC8303777

[27] Tagliafico AS, Piana M, Schenone D, Lai R, Massone AM, Houssami N . Overview of radiomics in breast cancer diagnosis and prognostication. Breast 2020; 49: 74–80. doi: 10.1016/j.breast.2019.10.018 31739125 PMC7375670

[28] Mezrich JL . Is artificial intelligence (AI) a pipe dream? why legal issues present significant hurdles to AI autonomy. AJR Am J Roentgenol 2022; 219: 152–56. doi: 10.2214/AJR.21.27224 35138133

